# Tebuconazole Induces Mouse Fetal Testes Damage via ROS Generation in an Organ Culture Method

**DOI:** 10.3390/ijms25137050

**Published:** 2024-06-27

**Authors:** Won-Young Lee, Ran Lee, Hyun-Jung Park

**Affiliations:** 1 Department of Livestock, Korea National University of Agriculture and Fisheries, Jeonju-si 54874, Republic of Korea; leewy81@korea.kr; 2 Department of Animal Biotechnology, College of Life Science, Sangji University, Wonju-si 26339, Republic of Korea; ranran2424@gmail.com

**Keywords:** tebuconazole, fetal testes, reactive oxygen species, germ cell

## Abstract

The fungicide tebuconazole (TEB) poses risks to human and animal health via various exposure routes. It induces toxicity in multiple organs and disrupts reproductive health by affecting steroid hormone synthesis and fetal development. In this study, we investigated the impact of TEB on fetal testes using in vitro models, focusing on germ, Sertoli, and Leydig cells, and explored the mechanisms underlying cellular damage. The results revealed significant damage to germ cells and disruption of Leydig cell development. TEB exposure led to a decrease in germ cell numbers, as indicated by histological and immunostaining analyses. TEB induced the up- and down-regulation of the expression of fetal and adult Leydig cell markers, respectively. Additionally, TEB-treated fetal testes exhibited increased expression of oxidative-stress-related genes and proteins. However, co-treatment with the antioxidant N-acetylcysteine mitigated TEB-induced germ cell damage and prevented abnormal Leydig cell development. These findings suggest that administration of antioxidants can prevent the intratesticular damage typically caused by TEB exposure.

## 1. Introduction

Tebuconazole (TEB) is a triazole fungicide widely used in agriculture to protect crops from fungal diseases. TEB inhibits the growth of fungi and disrupts their ability to reproduce, ultimately preventing disease spread in plants. Although it contributes to increased productivity in agriculture, it can be toxic to humans and animals [1].

Human and animal exposure to TEB can occur via various routes, including inhalation, skin contact, and ingestion. For example, individuals working with TEB in agricultural settings, such as farmers, agricultural workers, and pesticide applicators, may be exposed to fungicides via the inhalation of airborne particles or direct contact through skin exposure [2] during the mixing, loading, and application of TEB.

Numerous studies have showed that TEB is toxic to various animal organs. Ku et al. showed that TEB accumulates in heart tissues and induces energy imbalances that trigger metabolic abnormalities via the IRS1/AKT and PPARγ/RXRα pathways [3]. TEB also induces liver damage along with disturbances in hepatic metabolism mediated by reactive oxygen species (ROS) [4]. TEB stimulates apoptotic cell death via the mitochondrial pathway and ROS-mediated endoplasmic reticulum (ER) stress in HCT116 intestinal cells [5].

Several studies have reported that triazole fungicides negatively affect the reproductive system. Prochloraz and TEB are azole fungicides and share common characteristics, including the extension of gestational length in dams, virilization of female pups, and alterations in steroid hormone levels in fetuses and/or dams [6,7,8]. In utero exposure of pregnant rats to 50–100 mg/kg TEB is associated with reproductive developmental effects in their offspring. Specifically, critical enzymes associated with steroid hormone synthesis have shown disrupted activity rat offspring [6].

In bovine studies, TEB affected the physiological functions of testicular cells and epididymal spermatozoa. Although TEB did not affect spermatozoa motility and plasma membrane integrity, spermiotoxicity was observed when spermatozoa were treated with 1–100 µM TEB for 6 h [9]. Treatment of bovine luteal cells with 100 µM TEB for 5 days resulted in a 65% decrease in progesterone levels. Therefore, TEB negatively impacts both male testicular cells and female luteal steroidogenic cells [10].

In addition to its effects on mammals, TEB induces accelerated metamorphosis at low concentrations (0.001 mg/L), accompanied by increased mortality and significant differences in the larval weight of *Rhinella arenarum* [11]. Additionally, parental exposure to TEB induces thyroid endocrine disruption in both the F_0_ and F_1_ generations and leads to defects in neuronal and cardiovascular development in the offspring of zebrafish. TEB also causes an imbalance in steroid synthesis with reduced expression of *cyp19a*, consistent with decreased levels of E2 protein [12,13].

A recent study showed that pubertal and gestational exposure to TEB affects the development of rat testes, with effects limited to Leydig cells [14,15]. During the pubertal stage, rats were exposed to 25–100 mg/kg·day TEB for 21 days. The results showed that the levels of serum testosterone increased and those of estradiol decreased without affecting the levels of serum luteinizing hormone and follicle-stimulating hormone. In addition, the expression levels of *Cyp11a1*, *Hsd11b1*, and *Fshr*, which are related to Leydig cell development, were up-regulated in the testes but did not stimulate the proliferation of Leydig cells [14].

Another study on the effects of gestational exposure to TEB on the development of fetal Leydig cells (FLCs) in rats revealed that the number of FLCs and levels of testosterone were increased after TEB exposure. TEB also up-regulated AKT1/mTOR/ERK1/2 signaling, which led to reduced autophagy. However, these studies focused only on the development of FLCs and the results do not reflect the overall effects of TEB exposure on fetal testes [15]. In the current study, we examined the damage to germ cells and FLC development caused by exposure to TEB and the underlying mechanisms.

The biological action of TEB in vitro may slightly differ from that in vivo, as the endocrine system is a complex network of glands and organs that is difficult to replicate in vitro. Nevertheless, in vitro systems have various advantages, such as reduced ethical considerations and an easily controlled environment that allows for more precise analysis of the effects of specific conditions or treatments on the organ of interest. In addition, in vitro systems enable the study of complex cell–cell and cell–matrix interactions in a three-dimensional context. Particularly, experiments involving TEB can inflict pain on the animal under study; in vitro systems can greatly reduce the use of laboratory animals to assess reproductive toxicity.

In the present study, we investigated the effects of TEB on murine fetal testes using in vitro organ culture models. Specifically, we tested the effects of TEB on different testicular cell types, such as germ cells, Sertoli cells, and Leydig cells, to identify the mechanisms underlying cellular damage.

## 2. Results

### 2.1. TEB Induced Germ Cell Damage in Fetal Testes

**Figure 1:**
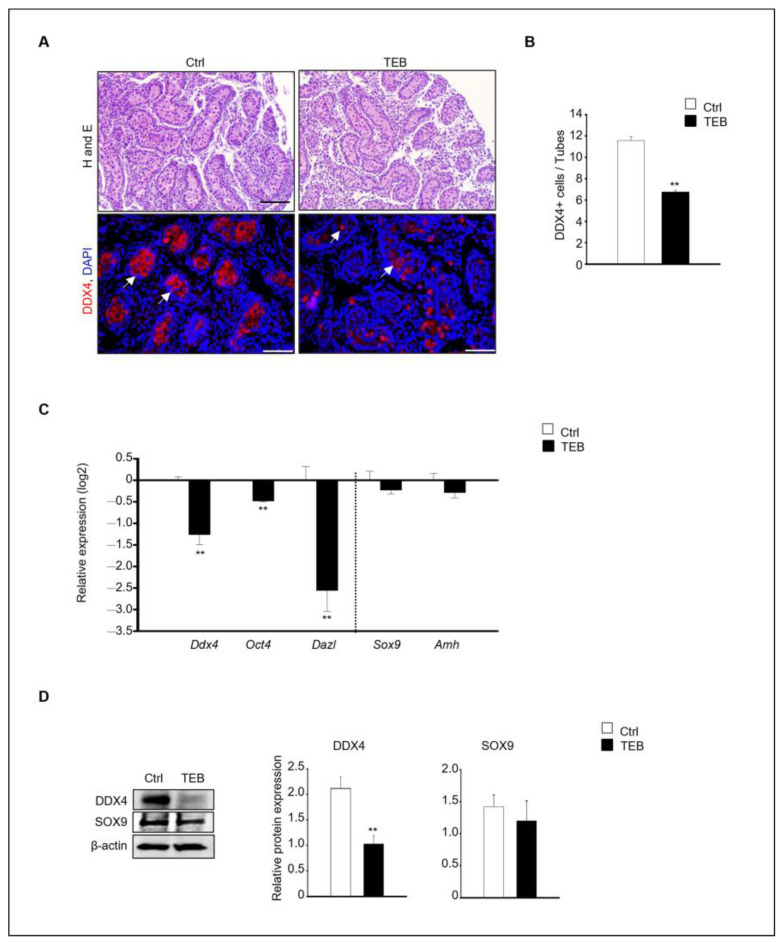
Effects of tebuconazole (TEB) on germ cells of fetal testes. (**A**) Hematoxylin and eosin staining for histological evaluation and TEB-exposed fetal testes subjected to immunostaining with anti-DDX4, a germ cell marker antibody. Germ cells showed red fluorescence (arrowhead). Scale bar = 200 μm. (**B**) Quantification of DDX4-positive cells. Data are shown as the mean ± standard error of the mean. (**C**) Quantitative real-time PCR (qPCR) detection of the gene expression of germ cell markers (*Ddx4*, *Oct4*, and *Dazl*) and Sertoli cell markers (*Sox9* and *Amh*) in the fetal testes. Data are presented as the mean ± standard error of the mean (*n* = 5) on a log_2_ scale. ** *p* < 0.01, compared with the control. (**D**) Protein expression of DDX4 and SOX9 in fetal testes cultured in the absence and presence of TEB (50 μM). Data are presented as the mean ± standard error of the mean (*n* = 3), with the relative quantification of protein expression. β-actin served as the loading control. ** *p* < 0.01, compared with the control.

To assess the impact of TEB on fetal testes development, 15.5 days post-coitum (dpc) fetal testes were cultured in vitro for 5 days and the histological alterations were examined. Histological differences between the control and TEB-treated groups were observed at the end of the culture. Numerous germ cells were present in the inner basement membrane of the seminiferous tubules in the control group, whereas fewer cells were observed in the TEB-treated group. For the precise definition of these histological changes, immunostaining was performed to detect the germ cell marker DEAD-box helicase 4 (DDX4) (Figure 1A). DDX4-positive cells were abundant in the control group but were relatively scarce (fewer BY 50%) in the TEB-treated group (Figure 1B). The gene expression of the germ cell markers *Ddx4, Oct4,* and *Dazl* and the Sertoli cell markers *Sox9* and *Amh* was examined in fetal testes exposed to TEB. The results demonstrated significant differences in germ cell markers between the control and TEB-exposed samples (*p* < 0.009). In contrast, Sertoli cell markers did not significantly differ between the two groups (*p* > 0.314).

In agreement with the immunostaining results, the expression of germ cell markers was significantly decreased following TEB treatment. In contrast, the expression of Sertoli cell markers was not significantly different between the control and TEB-treated groups (Figure 1C). The protein expression of DDX4 and SOX9, measured using immunoblotting, revealed that DDX4 levels were significantly reduced (*p* = 0.009), whereas SOX9 levels were not reduced in TEB-exposed fetal testes (*p* = 0.306) (Figure 1D).

### 2.2. Effects of TEB on Leydig Cell Development in Fetal Testes

**Figure 2:**
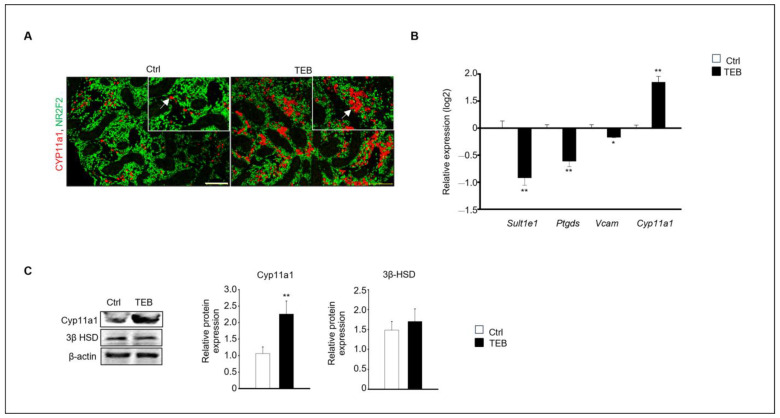
Expression of fetal (FLC) and adult Leydig cell (ALC) markers in a developing fetal testis. (**A**) Co-immunostaining of CYP11A1 (red, arrowhead), an FLC marker (green), and NR2F2, an interstitial marker, in fetal testes cultured in the absence and presence of TEB. The framed area shows a 1.5 × magnification of the original image. Scale bar = 200 μm. (**B**) Gene expression of FLC marker *Cyp11a1* and ALC markers *Sult1e1*, *Ptgds*, and *Vcam* in TEB-treated fetal testes is presented as the mean ± standard error of the mean (*n* = 5) on a log_2_ scale. (**C**) Protein expression of CYP11A1 in TEB-treated fetal testes. The relative protein expression levels are shown as the mean ± standard error of the mean (*n* = 3). Significant differences between untreated and TEB-treated groups are indicated by asterisks (*n* = 4 and * *p* < 0.05 and ** *p* < 0.01 compared with the control).

Two distinct types of Leydig cells are present during testes development: FLCs and adult Leydig cells (ALCs). FLCs are initially present in the embryonic testes until birth; however, their numbers rapidly decrease shortly afterward. In contrast, the number of ALCs increases during puberty [16]. We investigated the effect of TEB on the development of FLCs. *Cyp11a1*, a standard FLC marker, was detected using immunostaining, which revealed that *Cyp11a1*-positive cells were more abundant in the TEB-exposed fetal testes than in the control (Figure 2A). Gene expression of the ALC markers *Sult1e1*, *Ptgds*, and *Vcam* and the FLC marker *Cyp11a1* was analyzed using quantitative real-time PCR (qPCR). The results showed that the expression of *Cyp11a1* increased, whereas that of the ALC markers decreased in TEB-exposed fetal testes compared with that in the control (Figure 2B). Cyp11a1 protein expression significantly increased with TEB treatment, whereas the expression of 3β-HSD, detected in both FLCs and ALCs, did not significantly differ between TEB-exposed fetal testes and control (Figure 2C). Therefore, TEB impeded the normal development of FLCs.

### 2.3. TEB-Induced Oxidative Stress in Fetal Testes

**Figure 3:**
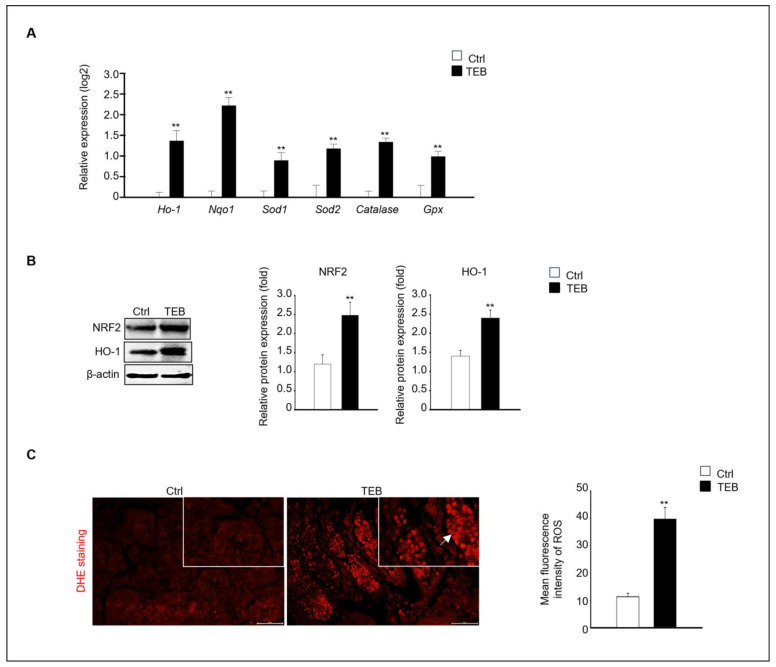
Expression of oxidative-stress-related genes and proteins. (**A**) Gene expression of *Ho-1*, *Nqo1*, *Sod1*, *Sod2*, *Cat*, and *Gpx* in TEB-treated and control groups. The relative gene expression is presented as the mean ± standard error of the mean (*n* = 4) on a log_2_ scale. ** *p* < 0.01 compared with the control. (**B**) NRF2 and HO-1 protein expression in each experimental group. Protein expression levels relative to that of β-actin are presented as the mean ± standard error of the mean (*n* = 4). (**C**) Immunofluorescence images of fetal testes evaluated using dihydroethidium (DHE) staining in each experimental group. DHE fluorescence (arrowhead) is presented as the mean gray value of DHE intensity obtained from 300 cells. The framed area shows a 1.5× magnification of the original image. The data are shown as the mean ± standard error of the mean (*n* = 5). Scale bar = 50 μm. ** *p* < 0.01 compared with controls.

Next, we investigated the mechanism of fetal testicular damage upon TEB exposure. The expression of oxidative-stress-related genes was assayed in cultured fetal testes following TEB treatment. Expression of *Ho-1*, *Nqo1*, *Sod1*, *Sod2*, *Cat*, and *Gpx* was significantly higher in TEB-exposed fetal testes than in the controls (Figure 3A). Similarly, the protein levels of NRF2 and HO-1 were higher in TEB-treated fetal testes than in the control (Figure 3B). Additionally, ROS production determined using dihydroethidium (DHE) staining was significantly higher in TEB-exposed fetal testes compared with that in controls (Figure 3C). DHE-positive cells were present in the seminiferous tubules of the fetal testes (Figure 3C).

### 2.4. N-Acetylcysteine Protects against TEB-Induced Testicular Damage

**Figure 4:**
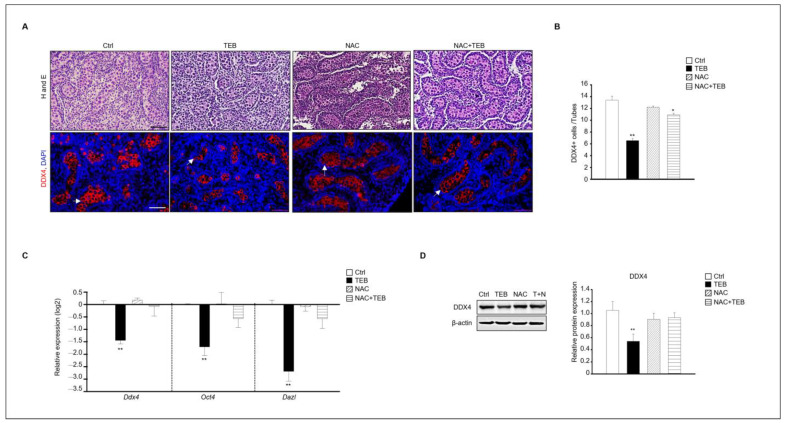
N-acetylcysteine (NAC) prevents germ cell damage caused by TEB treatment. (**A**) Histological analysis and immunostaining of DDX4 in fetal testes in each experimental group (Ctrl, TEB, NAC, and TEB+NAC). Arrowheads indicate DDX4-positive cells. DAPI-stained nucleus. Scale bar = 50 μm. (**B**) Quantification of DDX4-positive cells. Data are shown as the mean ± standard error of the mean. (**C**) Gene expression of *Ddx4*, *Oct4*, and *Dazl* in each group determined using qPCR. Data are presented as the mean ± standard error of the mean (*n* = 5). (**D**) Protein expression of DDX4 in each group. The level of DDX4 protein expression relative to that of β-actin is shown as the mean ± standard error of the mean (*n* = 3). * *p* < 0.05 and ** *p* < 0.01 compared with the control.

Based on the results shown in Figure 3, we found that the testicular damage caused by TEB involved ROS. Therefore, we investigated whether N-acetylcysteine (NAC), an antioxidant, could prevent the damage to fetal testes caused by TEB treatment. The results of immunostaining showed that the number of DDX4-positive germ cells was reduced in the group treated with TEB alone but not in the group treated with both TEB and NAC. Therefore, NAC prevented the germ cell damage caused by TEB. To verify this result, the gene expression of germ cell markers, such as *Ddx4*, *Oct4*, and *Dazl*, was evaluated. Expression of these genes was significantly decreased in the TEB-treated group but not in the NAC+TEB-treated group (Figure 4C). The protein expression of DDX4 was reduced in the TEB-treated group, whereas the expression was similar in the NAC+TEB-treated and control groups (Figure 4D).

**Figure 5:**
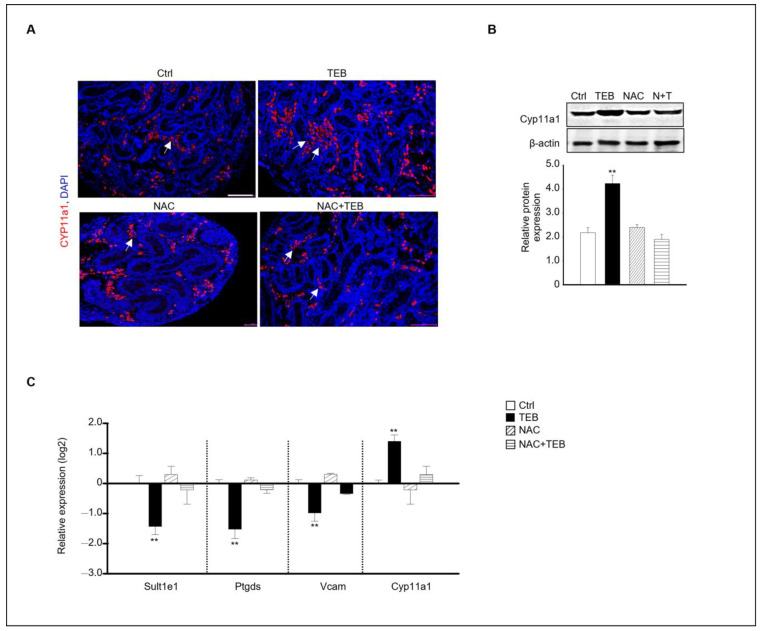
Effect of NAC on FLC development in TEB-exposed fetal testes. (**A**) Immunostaining of the FLC marker CYP11A1 in fetal testes in each experimental group (Ctrl, TEB, NAC, and TEB+NAC). Arrowhead indicates a cluster of FLCs. DAPI-stained nucleus. Scale bar = 200 μm. (**B**) CYP11A1 protein expression in each group detected using immunoblotting. The relative protein expression is presented as the mean ± standard error of the mean (*n* = 3). (**C**) The relative gene expression of *Sult1e1*, *Ptgds*, *Vcam*, and *Cyp11a1* determined using qPCR. Data are presented as the mean ± standard error of the mean (*n* = 5). ** *p* < 0.01 compared with the control.

Our results indicated that TEB prevented Leydig cell development in the fetal testes. Based on this finding, we investigated whether NAC treatment could prevent the inhibition of the normal development of Leydig cells caused by TEB in the fetal testes. Immunostaining with *Cyp11a1*, an FLC marker, showed that *Cyp11a1*-positive FLCs were abundant in the TEB-treated group but not in the TEB+NAC-treated group (Figure 5A). Similar to the staining result, the protein expression of Cyp11a1 was significantly increased in TEB-treated samples but not in NAC+TEB-treated samples (Figure 5B). To support the protein expression data, the gene expression of FLC and ALC markers was evaluated. The expression of *Cyp11a1* was similar to that in the control group and the expression of the ALC markers *Sult1e1*, *Ptgds*, and *Vcam* was up-regulated in the co-treated groups (Figure 5C).

### 2.5. NAC Prevents the Increase in Oxidative Stress Caused by TEB in Fetal Testes

**Figure 6:**
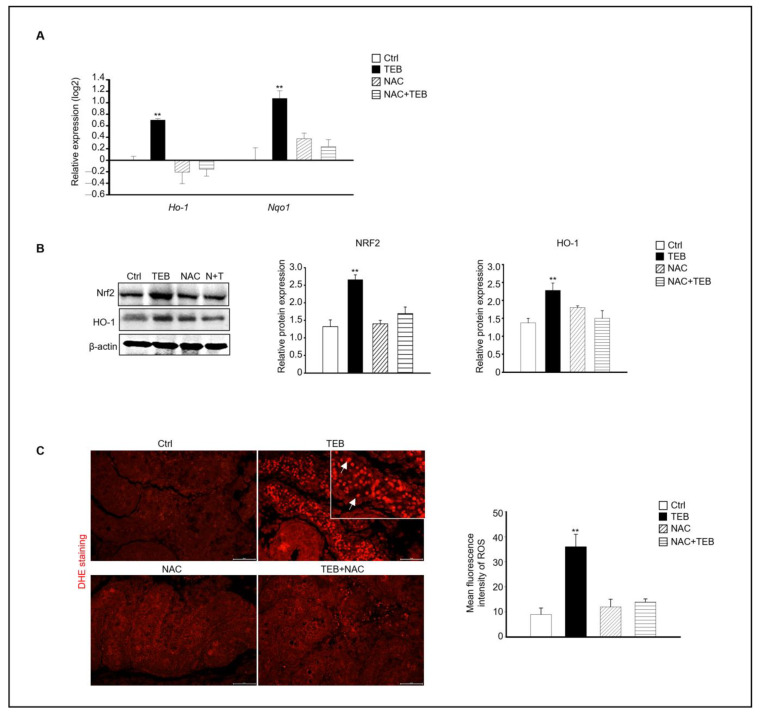
Effect of NAC on TEB-induced reactive oxygen species (ROS) in fetal testes. (**A**) Gene expression of *Ho-1* and *Nqo1* in fetal testes in each experimental group. The data are presented as the mean ± standard error of the mean (*n* = 4). (**B**) Protein expression of NRF2 and HO-1 relative to that of β-actin in each sample, presented as the mean ± standard error of the mean (*n* = 3). (**C**) Dihydroethidium (DHE)-positive cells (arrowhead) in the testes. Mean fluorescence intensity of ROS in each sample. The framed area shows a 1.5× magnification of the original image. Data are shown as the mean ± standard deviation (*n* = 3). ** *p* < 0.01 compared with the control.

Based on our results, the expression of the ROS-production-related genes *Ho-1* and *Nqo1*, which are up-regulated by oxidative stress, was evaluated in each experimental group. Similar to previous results, the TEB-treated group showed increased expression of *Ho-1* and *Nqo1* compared with that in the control group, whereas the NAC+TEB-treated group showed decreased expression compared with that in the TEB-treated group (Figure 6A). The protein expression of both NRF2 and HO-1 was higher the TEB-treated group than in the control group and similar in the control and TEB+NAC groups (Figure 6B). DHE staining of the fetal testes showed that the DHE-positive cell population was significantly high in the TEB-treated group but not in the other groups. These results indicate that ROS production was suppressed by NAC in the seminiferous tubules of the fetal testes (Figure 6C).

## 3. Discussion

TEB is effective against a wide range of fungal diseases that affect a variety of crops, such as cereals, fruits, and vegetables. It helps prevent yield losses and maintains crop quality by controlling fungal pathogens [17]. However, TEB is associated with risks such as human health concerns, residue accumulation in various organisms, and ecological impacts [3,4]. We focused on the toxic effects of TEB during the early stages of testes development and found that the number of germ cells was significantly reduced in TEB-exposed fetal testes. In support of our findings, a previous study showed that gestational exposure to TEB affects the development of rat FLCs; however, the effects on germ cells in the testes were not evaluated [15]. TEB exposure for 10 days starting on day 12 of pregnancy led to increased levels of fetal serum testosterone and progesterone and an increased number of FLCs per testis without the induction of cell aggregation. In addition, TEB up-regulated the expression of steroidogenesis-related genes, such as *Star*, *Cyp11a1*, and *Hsd17b3*, in the fetal testes. In primary Leydig cells derived from fetal testes at gestation day 21, TEB treatment resulted in an increased phosphorylation of AKT1, ERK1/2, and mTOR and decreased the levels of Beclin1, LC3A/B, and BAX, which are related to autophagy and proliferation [5]. Similarly, we showed that *Cyp11a1*-positive FLCs were more abundant in TEB-treated than in untreated fetal testes. In contrast, the expression of ALC markers, including *Sult1e1*, *Ptgds*, and *Vcam*, was significantly decreased in the TEB-treated group compared with that in the control group. Exposure to TEB resulted in observable damage to germ cells and an imbalance in FLC development. However, TEB exposure did not affect fetal Sertoli cells in the testes, as the expression of *Sox9* and *Amh* did not differ from that in the non-exposed group. Similarly, Ma et al. reported that *Sox9* expression in fetal testes did not change after exposure to TEB [15]. TEB is an endocrine-disrupting chemical; therefore, most studies have focused on Leydig cells, which are involved in hormone synthesis. Another research group investigated the effects of TEB in testes until the pubertal stage of rats and showed an increase in testosterone production via inhibition of aromatase activity after TEB administration. Interestingly, in the pubertal stage, TEB did not affect the number of *Cyp11A1*-positive Leydig cells, which differs from the results of our study [14]. During FLC development, their numbers rapidly decline after birth. These cells are expected to undergo either apoptosis or degeneration and are eventually replaced by newly formed ALCs in the postnatal testes [18,19]. Therefore, we conducted an expression analysis to distinguish between FLC and ALC markers in TEB-exposed fetal testes. These markers were confirmed based on our previous study, in which we screened for changes in their gene expression from 15.5–19.5 dpc during fetal testes development [20].

A reduction in DDX4-positive germ cell numbers following TEB exposure may result from the direct effects of TEB or indirectly by causing abnormalities in FLC development. Although toxicity studies have not been performed using early-stage germ cells following TEB exposure, a study reported that TEB accumulated in the gonads and that cumulative egg production was clearly reduced in TEB-exposed zebrafish [21]. In the present study, we observed a decrease in the number of early-stage germ cells following TEB exposure.

The molecular mechanisms underlying the effects of TEB exposure within cells and tissues are under active research. Othmène et al. found that in human colorectal carcinoma cells (HCT 116), TEB triggered mitochondria-mediated apoptosis via the ER stress pathway and induced ROS generation [5]. In one of our previous studies, using a bovine mammary epithelial cell line (MAC-T cell), we showed that TEB induced ER-stress-mediated cell death via up-regulation of Bip/GRP78, PDI, ATF4, CHOP, and ERO1-Lα [22]. In addition, Li et al. described that TEB exerted hepatotoxic effects in adult and larval zebrafish by inducing oxidative stress. The activities of total superoxide dismutase (T-SOD), catalase, peroxidase, and glutathione *S*-transferase (GST) were significantly increased in the livers of male and female zebrafish [23]. Another study demonstrated the cytotoxic and inflammatory effects of TEB and econazole in TM4 cells, which are murine Sertoli cells. They showed an increase in ROS production, decrease in SOD and GST activity, and increase in oxidized glutathione levels. In addition, COX-2 expression was induced and TNF-α production was increased [24]. Based on these studies, we also investigated testicular damage via ROS in fetal testes following TEB exposure. We found that the expression of *Ho-1*, *Nqo1*, *Sod1*, *Sod2*, *catalase*, and *Gpx* was up-regulated in TEB-exposed fetal testes compared with that in the control testes. *Nrf2*/HO-1 signaling is a primary regulatory pathway for intracellular defense against oxidative stress. *Nrf2*, under the regulation of *Keap1*, manages oxidative stress. Under normal conditions, *Nrf2* is inactive in the cytoplasm and bound to *Keap1*. Upon oxidative stress, *Nrf2* is activated and translocated to the nucleus, where it stimulates the transcription of cytoprotective genes via the antioxidant response element. This gene expression enhances cellular defenses against oxidative stress and foreign substrates [25,26]. Chen et al. investigated the protection conferred by the *Nrf2-Keap1* pathway against the detrimental effects of oxidative stress upon treatment with deltamethrin (DM), a stress inducer and type of pesticide. They showed that DM promoted the up-regulation of *Nrf2* expression and nucleation via oxidative stress in *Drosophila* Kc cells, similar to the results of our study [27]. *Nrf2*-mediated transcriptional processes initiate the regulation of downstream gene expression, activating a cascade of antioxidant enzymes including HO-1, NQO1, SOD, and glutathione peroxidase. This cascade works to eliminate ROS and other harmful substances, promoting antioxidative stress, anti-inflammatory, anti-apoptotic, and other cytoprotective mechanisms [28,29]. In the present study, we observed that various factors related to ROS production were increased in TEB-exposed fetal testes. The fetal testes were co-treated with an antioxidant NAC to verify these findings. The number of DDX4-positive cells in the testes was similar between the co-treated and untreated groups. Additionally, there was a decrease in the number of *Cyp11a1*-positive FLCs, which were increased after TEB exposure. Moreover, the expression levels of *Ho-1* and *Nqo1* in the NAC+TEB-treated group were similar to those in the control group, whereas they were increased in the TEB-treated group.

Interestingly, DHE-positive cells were detected in the seminiferous tubules of the fetal testes, indicating that ROS production occurs in the germ cells. Although ROS-mediated cellular damage occurs in germ cells present in the seminiferous tubules, Leydig cell development was normal in the testes after NAC treatment. Another study revealed the presence of germ-cell-derived signals that indirectly or directly affect other testicular cells, including Sertoli and Leydig cells. The absence of germ cells leads to the up-regulation of numerous genes in both Sertoli and Leydig cells, suggesting that germ cells play crucial roles in the structure and function of the mouse testes. Furthermore, testes lacking germ cells may exhibit fluctuations in hormone production, particularly during fetal development. Excessive testosterone production can affect the masculinization of various tissues [30,31]. Various events that occur in germ cells likely influence the normal development of Leydig cells.

Regarding TEB accumulation in body, several studies have reported the detection of TEB residues in the urine of both children and adults, with median levels between 15.6 and 27.6 µg/L (50.8–90 nM); 143.1 ng/g TEB was detected in the hair of an agricultural worker [32,33,34]. Approximately 200–1600 µg/kg TEB has been observed in vegetables and soil [35,36]. Although the concentration of TEB used in this study was higher than that used in previous studies, cellular damage following TEB exposure is thought to vary widely depending on the exposure time and environment.

Taken together, developmental disorders in FLCs and decreased germ cell numbers in the fetal testes may be attributed to the physiological changes induced by oxidative stress.

## 4. Materials and Methods

### 4.1. Organ and Cell Cultures

Pregnant CD-1 female mice at 10.5 dpc were acquired from Dae Han Bio Link Co. (Daejeon, Republic of Korea) and underwent an additional 4 days of breeding in cages to obtain fetal testes cultures. The mice were maintained under a 12 h photoperiod at an ambient temperature range of 20–25 °C. All experimental procedures were approved by the Institutional Animal Care and Use Committee of Sangji University (Registration No. 2023-10). Pregnant mice were euthanized at 15.5 dpc, and 250 mg/kg of avertin was used to harvest the testes from male fetuses. Organ cultures of the fetal testes were performed as described previously [20]. Briefly, decapsulated fetal testes were positioned on Millicell inserts (Millipore, Etobicoke, ON, Canada) and cultured in medium (DMEM/F-12, 15 mM HEPES, and 1 × gentamicin and amphotericin B; Invitrogen, Carlsbad, CA, USA) with or without TEB (50 μM). The cultures were incubated at 34 °C under 5% CO_2_, with daily medium changes over a 5-day period. TEB and NAC were dissolved in dimethyl sulfoxide and diluted in the culture medium to achieve final concentrations of 50 μM TEB and 5 mM NAC. The final concentrations were determined based on previous studies and Figure S1 [37,38,39].

### 4.2. Histological Analysis and Immunostaining

The cultured testicular samples were fixed in 4% paraformaldehyde for 2 h, followed by gradual dehydration, paraffin embedding, and microtome sectioning (5 μm thick sections) using a Leica Biosystems microtome (Nussloch, Germany). The sections were stained with hematoxylin and eosin. Five distinct testicular areas were examined by a blinded analysis using a microscope. Immunostaining was performed as previously described [20] and the stained samples were observed under a light microscope (Olympus IX73, Shinjuku-ku, Tokyo, Japan). Protein expression was quantified in at least four samples from each experimental group. For each sample, testicular cross sections with a minimum of 40 tubules were scored for analysis. Detailed information regarding the primary antibodies used in this study is provided in Table 1.

### 4.3. Western Blotting

Proteins for Western blot analysis were extracted using RIPA buffer (Thermo Fisher Scientific, Waltham, MA, USA) containing a protease inhibitor cocktail (Roche, Mannheim, Germany). The extracted proteins (each 30 μg) were loaded onto 4–20% acrylamide gels (Bio-Rad, Hercules, CA, USA) and transferred to a polyvinylidene fluoride (PVDF) membrane. The PVDF membrane was then incubated for 16 h at 4 °C with a primary antibody. Table 1 depicts the details regarding the primary antibodies used in this study. The PVDF membrane was exposed to a secondary antibody for 1 h at 24 °C. Protein bands were visualized using a West Pico chemiluminescent substrate (Thermo Fisher Scientific, Rockford, IL, USA), and the blot images were captured on an iBright™ Imaging System (Thermo Fisher Scientific). The anti-β-actin antibody served as a control for normalizing the expression of the protein of interest. Table 2 shows the antibodies used for immunoblotting.

### 4.4. qPCR Analysis

RNA was extracted using an RNeasy Mini Kit with on-column DNase treatment (Qiagen, Hilden, Germany). cDNA was generated using reverse transcription with the RevertAid First Strand cDNA Synthesis Kit (Thermo Fisher Scientific). qPCR was performed on a QuantStudio 1 instrument (Applied Biosystems, Foster City, CA, USA) using a SYBR mixture (Thermo Fisher Scientific). The reaction conditions and data analyses were in accordance with our previous studies [20,40]. Quantification was performed by normalizing to endogenous GAPDH levels. The data are presented on a log_2_ scale. The primers used in the experiments, including those designed using Primer3 software (http://frodo.wi.mit.edu accessed on 3 June 2021), are listed in Table 3.

### 4.5. DHE Fluorescence Staining

The ROS levels in fetal testes were determined using DHE (red) fluorescence, as previously described [41]. Briefly, after deparaffinization, the testis sections were incubated with a 10 μM DHE solution for 60 min at 37 °C. Stained sections were washed with water and visualized under an Olympus IX73 microscope.

### 4.6. Statistical Analysis

The data are presented as the mean ± standard error of the mean, based on a minimum of three independent experiments, each conducted with triplicate samples. Differences in the means were calculated using one-way analysis of variance, followed by Tukey’s post-hoc test. Statistical analyses were performed using the SPSS statistical package (version 15.0) for Windows (IBM, Somers, NY, USA). Significant differences between groups are indicated by one asterisk (*) for *p* < 0.05 and two asterisks (**) for *p* < 0.01.

## 5. Conclusions

We investigated the molecular mechanisms underlying TEB toxicity in fetal testes cultures in vitro. TEB significantly reduced the number of germ cells and expression levels of their marker genes; however, the expression of Sertoli cell marker genes remained unchanged. In addition, although FLCs were abundant, the expression of ALC marker genes was down-regulated in TEB-treated fetal testes. Moreover, damage to fetal testes by TEB was suppressed by antioxidant treatment. We focused on the cell types present in the fetal testes (germ cells, FLCs, and ALCs). Further experiments are required to evaluate other cell types to clarify the mechanisms underlying TEB cytotoxicity. Our study is the first to evaluate the germ cells damage caused by TEB and its underlying mechanism.

## Figures and Tables

**Table 1:** Antibodies used for immunostaining.

Antibody	Company	Catalog Number	Dilution
DDX4	Abcam (Waltham, MA, USA)	ab13840	1:300
NR2F2	Abcam	ab41859	1:300
CYP11A1	Cell Signaling Technology (Danvers, MA, USA)	#14217	1:200

**Table 2:** Antibodies used for immunoblotting.

Antibody	Company	Catalog Number	Dilution
DDX4	Abcam	ab13840	1:1000
SOX9	Abcam	ab185230	1:1000
NRF2 HO-1	Santa Cruz Biotechnology (Dallas, TX, USA) Santa Cruz Biotechnology	sc365949 sc136960	1:100 1:1000
CYP11A1	Cell Signaling Technology	#14217	1:1000
3β-HSD	Santa Cruz Biotechnology	sc30820	1:1000
ACTB	Santa Cruz Biotechnology	ab13840	1:1000

**Table 3:** Primers used for reverse transcription–polymerase chain reaction using mouse cDNA.

Gene	Forward Primer	Reverse Primer
*Dazl*	5′-GTCGAAGGGCTATGGATTTG-3′	5′-ACGTGGCTGCACATGATAAG-3′
*Ddx4*	5′-CCGCATGGCTAGAAGAGATT-3′	5′-TTCCTCGTGTCAACAGATGC-3′
*Oct4*	5′-TGAGGCTACAGGACACCTTTC-3′	5′-GTGCCAAAGTGGGGACCT-3′
*Sox9*	5′-AGTACCCGCATCTGCACAAC-3′	5′-TACTTGTAATCGGGGTGGTCT-3′
*Amh*	5′-GGGCCTCATCTTAACCCTTC-3′	5′-AGTCATCCGCGTGAAACAG-3′
*Cyp11a1*	5′-GACAATGGTTGGCTAAACCTG-3′	5′-GGGTCCACGATGTAAACTGAC-3′
*Sult1e1*	5′-AAAGGGAATTATAGGAGACTGGA-3ʹ	5′-TGCTGCTTGTAGTGCTCATCAA-3′
*Ptgds*	5′-GCTCTGAGCAAATGGCTGC-3′	5′-GGGAATCCCAAGAGACCCAG-3′
*V* *cam1*	5′-TGCCGAGCTAAATTACACATTG-3′	5′-CCTTGTGGAGGGATGTACAGA-3′
*Gapdh*	5′-GTCGGTGTGAACGGATTTG-3′	5′-CTTGCCGTGGGTAGAGTCAT-3′

## Data Availability

Data will be made available on request.

## Supplementary Materials

The following supporting information can be downloaded at: https://www.mdpi.com/article/10.3390/ijms25137050/s1.

## Abbreviations

TEB	Tebuconazole
OCT4	Octamer-binding transcription factor 4
NAC	N-Acetylcysteine
IRS1	Insulin receptor substrate 1
AKT	AKT Serine/threonine kinase
PPARγ	Peroxisome proliferator-activated receptor gamma
RXRα	Retinoid X receptor
ER	Endoplasmic reticulum
E2	Estradiol
Cyp11a1	Cytochrome P450 family 11 subfamily A member 1
Hsd11b1	Hydroxysteroid 11-beta dehydrogenase 1
Fshr	Follicle stimulating hormone receptor
mTOR	Mammalian target of rapamycin
ERK1/2	Extracellular signal-regulated protein kinases 1 and 2
DDX4	DEAD-box helicase 4
3βHDS	3β-Hydroxysteroid dehydrogenase/Δ5-4 isomerase
SOX9	SRY-box transcription factor 9
Dazl	Deleted in azoospermia like
Amh	Anti-Müllerian hormone
ALCs	Adult Leydig cells
FLCs	Fetal Leydig cells
Sult1e1	Sulfotransferase 1E1
Ptgds,	Prostaglandin D_2_ synthase
Vcam,	Vascular cell adhesion molecule 1
Ho-1	Heme oxygenase 1
SOD	Superoxide dismutase
Gpx	Glutathione peroxidase
NRF2	Nuclear factor erythroid 2–related factor 2
DHE	Dihydroethidium
LC3A/B	Microtubule-associated protein 1 light chain 3 alpha/beta
BAX	BCL2-associated X, apoptosis regulator
GRP78	Glucose-regulated protein 78
PDI	Protein disulfide isomerase
ATF4	Activating transcription factor 4
CHOP	C/EBP homologous protein 10
ERO1-Lα	Endoplasmic reticulum oxidoreductase 1 alpha
GST	Glutathione *S*-transferase
COX-2	Cyclooxygenase 2
Keap1	Kelch-like ECH-associated protein 1
NQO1	NAD(P)H dehydrogenase 1

## References

[1] Strickland T.C., Potter T.L., Joo H. (2004). Tebuconazole dissipation and metabolism in Tifton loamy sand during laboratory incubationt. Pest. Manag. Sci..

[2] Rosa M.J., Armendáriz-Arnez C., Gudayol-Ferré E., Prehn M., Fuhrimann S., Eskenazi B., Lindh C.H., Mora A.M. (2024). Association of pesticide exposure with neurobehavioral outcomes among avocado farmworkers in Mexico. Int. J. Hydrogen Environ. Health.

[3] Ku T., Hu J., Zhou M., Xie Y., Liu Y., Tan X., Guo L., Li G., Sang N. (2024). Cardiac energy metabolism disorder mediated by energy substrate imbalance and mitochondrial damage upon tebuconazole exposure. J. Environ. Sci..

[4] Ku T., Zhou M., Hou Y., Xie Y., Li G., Sang N. (2021). Tebuconazole induces liver injury coupled with ROS-mediated hepatic metabolism disorder. Ecotoxicol. Environ. Saf..

[5] Ben Othmène Y., Monceaux K., Belhadef A., Karoui A., Ben Salem I., Boussabbeh M., Abid-Essefi S., Lemaire C. (2022). Triazole fungicide tebuconazole induces apoptosis through ROS-mediated endoplasmic reticulum stress pathway. Environ. Toxicol. Pharmacol..

[6] Taxvig C., Hass U., Axelstad M., Dalgaard M., Boberg J., Andeasen H.R., Vinggaard A.M. (2007). Endocrine-Disrupting Activities In Vivo of the Fungicides Tebuconazole and Epoxiconazole. Toxicol. Sci..

[7] Vinggaard A.M., Nellemann C., Dalgaard M., Jørgensen E.B., Andersen H.R. (2002). Antiandrogenic effects in vitro and in vivo of the fungicide prochloraz. Toxicol. Sci..

[8] Vinggaard A.M., Christiansen S., Laier P., Poulsen M.E., Breinholt V., Jarfelt K., Jacobsen H., Dalgaard M., Nellemann C., Hass U. (2005). Perinatal exposure to the fungicide prochloraz feminizes the male rat offspring. Toxicol. Sci..

[9] Kabakci R., Kaya A., Yigit A.A., Varisli O. (2021). Assessment of tebuconazole exposure on bovine testicular cells and epididymal spermatozoa. Acta Vet. Hung..

[10] Atmaca N., Arikan S., Essiz D., Kalender H., Simsek O., Bilmen F.S., Kabakci R. (2018). Effects of mancozeb, metalaxyl and tebuconazole on steroid production by bovine luteal cells in vitro. Environ. Toxicol. Pharmacol..

[11] Acquaroni M., Cresto F.N., Pérez Coll C., Svartz G. (2024). Toxicity assessment of a tebuconazole-based fungicide on the embryo-larval development of the common south American toad Rhinella arenarum. Environ. Toxicol..

[12] Li S., Wu Q., Sun Q., Coffin S., Gui W., Zhu G. (2019). Parental exposure to tebuconazole causes thyroid endocrine disruption in zebrafish and developmental toxicity in offspring. Aquat. Toxicol..

[13] Li S., Sun Q., Wu Q., Gui W., Zhu G., Schlenk D. (2019). Endocrine disrupting effects of tebuconazole on different life stages of zebrafish (*Danio rerio*). Environ. Pollut..

[14] Chen X., Zhu Q., Li X., Huang T., Wang S., Wang Y., Chen X., Lin Z., Ge R.S. (2019). Pubertal exposure to tebuconazole increases testosterone production via inhibiting testicular aromatase activity in rats. Chemosphere.

[15] Ma F., Li Y., Yu Y., Li Z., Lin L., Chen Q., Xu Q., Pan P., Wang Y., Ge R.S. (2021). Gestational exposure to tebuconazole affects the development of rat fetal Leydig cells. Chemosphere.

[16] Griswold S.L., Behringer R.R. (2009). Fetal Leydig cell origin and development. Sex Dev..

[17] Berenzen N., Hümmer S., Liess M., Schulz R. (2003). Pesticide peak discharge from wastewater treatment plants into streams during the main period of insecticide application: Ecotoxicological evaluation in comparison to runoff. Bull. Environ. Contam. Toxicol..

[18] Lording D.W., De Kretser D.M. (1972). Comparative ultrastructural and histochemical studies of the interstitial cells of the rat testis during fetal and postnatal development. J. Reprod. Fertil..

[19] Shima Y. (2019). Development of fetal and adult Leydig cells. Reprod. Med. Biol..

[20] Park H.J., Lee W.Y., Do J.T., Park C., Song H. (2021). Evaluation of testicular toxicity upon fetal exposure to bisphenol A using an organ culture method. Chemosphere.

[21] Yan W., Li G., Lu Q., Hou J., Pan M., Peng M., Peng X., Wan H., Liu X., Wu Q. (2023). Molecular Mechanisms of Tebuconazole Affecting the Social Behavior and Reproduction of Zebrafish. Int. J. Environ. Res. Public Health.

[22] Lee W.Y., Lee R., Park H.J. (2023). Tebuconazole Induces ER-Stress-Mediated Cell Death in Bovine Mammary Epithelial Cell Lines. Toxics.

[23] Li S., Jiang Y., Sun Q., Coffin S., Chen L., Qiao K., Gui W., Zhu G. (2020). Tebuconazole induced oxidative stress related hepatotoxicity in adult and larval zebrafish (*Danio rerio*). Chemosphere.

[24] Petricca S., Carnicelli V., Luzi C., Cinque B., Celenza G., Iorio R. (2023). Oxidative Stress, Cytotoxic and Inflammatory Effects of Azoles Combinatorial Mixtures in Sertoli TM4 Cells. Antioxidants.

[25] Kim S.J., Park C., Lee J.N., Lim H., Hong G.Y., Moon S.K., Lim D.J., Choe S.K., Park R. (2015). Erdosteine protects HEI-OC1 auditory cells from cisplatin toxicity through suppression of inflammatory cytokines and induction of Nrf2 target proteins. Toxicol. Appl. Pharmacol..

[26] Misra J.R., Lam G., Thummel C.S. (2013). Constitutive activation of the Nrf2/Keap1 pathway in insecticide-resistant strains of Drosophila. Insect. Biochem. Mol. Biol..

[27] Chen L., Zhang T., Ge M., Liu Y., Xing Y., Liu L., Li F., Cheng L. (2020). The Nrf2-Keap1 pathway: A secret weapon against pesticide persecution in Drosophila Kc cells. Pestic. Biochem. Physiol..

[28] Cui W., Leng B., Wang G. (2019). Klotho protein inhibits H2O2-induced oxidative injury in endothelial cells via regulation of PI3K/AKT/Nrf2/HO-1 pathways. Can. J. Physiol. Pharmacol..

[29] Zhao C., Zhang Y., Liu H., Li P., Zhang H., Cheng G. (2017). Fortunellin protects against high fructose-induced diabetic heart injury in mice by suppressing inflammation and oxidative stress via AMPK/Nrf-2 pathway regulation. Biochem. Biophys. Res. Commun..

[30] Rios-Rojas C., Spiller C., Bowles J., Koopman P. (2016). Germ cells influence cord formation and Leydig cell gene expression during mouse testis development. Dev. Dyn..

[31] McCarthy M.M., Arnold A.P. (2011). Reframing sexual differentiation of the brain. Nat. Neurosci..

[32] Rosa M., Elisa P., Federico M.R., Stefan M.R., Andrea V., Claudio C., Angelo M., Silvia F. (2019). Assessment of penconazole exposure in winegrowers using urinary biomarkers. Environ. Res..

[33] Oerlemans A., Van Dael M.F.P., Vermeulen R.C.H.V., Russel F.G.M., Scheepers P.T.J. (2018). Urine collection methods for non-toilet-trained children in biological monitoring studies: Validation of a disposable diaper for characterization of tebuconazole exposure. Toxicol. Lett..

[34] Elisa P., Rosa M., Silvia F. (2018). Determination of tebuconazole and penconazole fungicides in rat and human hair by liquid chromatography/tandem mass spectrometry. Rapid Commun. Mass Spectrom..

[35] Wang Y., Huang T., Zhang T., Ma X., Zhou G., Chi M., Geng X., Yuan C., Zou N. (2023). Residue Levels and Dietary Intake Risk Assessments of 139 Pesticides in Agricultural Produce Using the m-PFC Method Based on SBA-15-C18 with GC-MS/MS. Molecules.

[36] Polat B., Tiryaki O. (2023). Determination of fungicide residues in soil using QuEChERS coupled with LC-MS/MS, and environmental risk assessment. Environ. Monit. Assess..

[37] Kim J.H., Park S.J., Kim T.S., Kim J.M., Lee D.S. (2016). Testosterone production by a Leydig tumor cell line is suppressed by hyperthermia-induced endoplasmic reticulum stress in mice. Life Sci..

[38] Liu J., Liu Q., Han J., Feng J., Guo T., Li Z., Min F., Jin R., Peng X. (2021). N-Acetylcysteine Inhibits Patulin-Induced Apoptosis by Affecting ROS-Mediated Oxidative Damage Pathway. Toxins.

[39] Zhang J., Lan T., Han X., Xu Y., Liao L., Xie L., Yang B., Tian W., Guo W. (2021). Improvement of ECM-based bioroot regeneration via N-acetylcysteine-induced antioxidative effects. Stem Cell Res. Ther..

[40] Lee W.Y., Park H.J. (2023). T-2 mycotoxin Induces male germ cell apoptosis by ROS-mediated JNK/p38 MAPK pathway. Ecotoxicol. Environ. Saf..

[41] Cho K.H., Nam H.S., Bahuguna A., Kim J.E. (2024). Long-Term Supplementation of Royal Jelly (Raydel^®^) Improves Zebrafish Growth, Embryo Production and Survivability, Blood Lipid Profile and Functionality of Vital Organs: A 72-Weeks’ Consumption Study. Pharmaceuticals.

