# Supplementary material for: Tebuconazole Induces Mouse Fetal Testes Damage via ROS Generation in an Organ Culture Method
Source: Int J Mol Sci. 2024 Jun 27;25(13):7050. doi: 10.3390/ijms25137050 (PMC11241142; doi:10.3390/ijms25137050)
Supplement: Supplementary file 1 [file ijms-25-07050-s001.zip › ijms-3045788-supplementary.pdf]

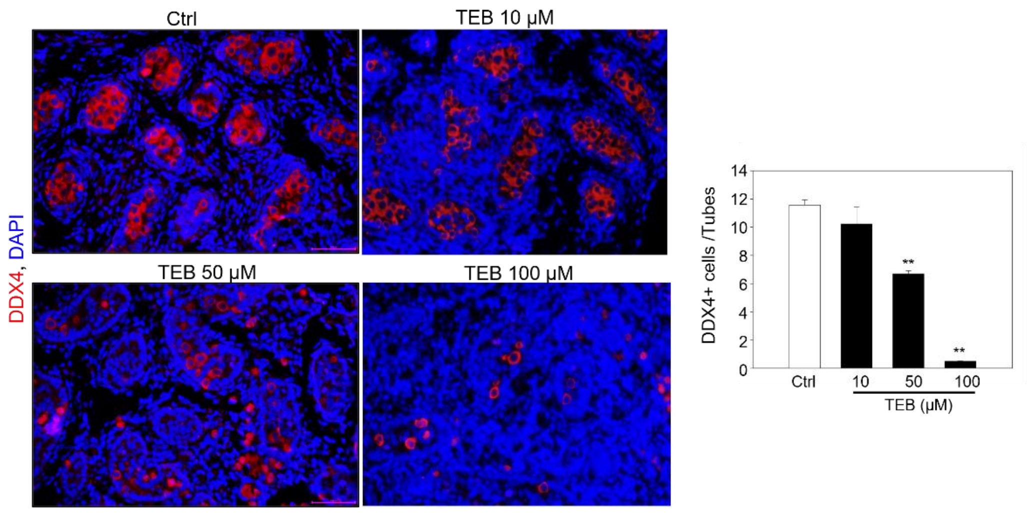

Figure S1. Immunostaining of DDX4 in 0-100 μM TEB exposed fetal testis. Graph showed mean and standard error of the mean (n = 4). Scale bar = 200 μm. ** $P < 0.01$ compared to controls.
